# A clinical analysis of nine new pediatric and adolescent cases of benign minor salivary gland neoplasms and a review of the literature

**DOI:** 10.1186/1752-1947-6-287

**Published:** 2012-09-11

**Authors:** Priyanshi Ritwik, Robert B Brannon

**Affiliations:** 1 Department of Pediatric Dentistry, LSU School of Dentistry, 1100 Florida Avenue, New Orleans, LA, 70119, USA; 2 Division of Oral and Maxillofacial Pathology, LSU School of Dentistry, 1100 Florida Avenue, New Orleans, LA, 70119, USA

**Keywords:** Benign tumors, Minor salivary gland, Benign tumors, Pediatric

## Abstract

**Introduction:**

Minor salivary gland neoplasms of epithelial origin are rare in children and adolescents and most are not well documented, except for a few small series and case reports. This study represents a retrospective clinical analysis of nine cases of benign epithelial salivary gland neoplasms accessioned over a 35-year period at the Louisiana State University School of Dentistry and combines the data with well-documented cases from the English-language literature.

**Methods:**

A retrospective clinical analysis of nine cases of benign epithelial salivary gland neoplasms was performed over a 35-year period at the Louisiana State University School of Dentistry and combined with data of well-documented cases from the English-language literature.

**Results:**

The nine benign salivary gland neoplasms in patients aged 19 months to 18 years accounted for 2.3% of the Louisiana State University School of Dentistry accessioned salivary gland tumors. These nine cases comprised eight pleomorphic adenomas and one cystadenoma. There were 40 cases in the literature, of which 34 were pleomorphic adenomas. Combining the data for the 42 pleomorphic adenomas resulted in a mean age of 12 years with a 2.8:1 female predilection. The hard palate and/or soft palate were the most common site (69.1%). The average duration and size was 2.1 years and 2.4cm, respectively. Bone involvement occurred in seven cases. Wide local excision was the treatment most often employed. Cases followed for two years or more had a recurrence rate of 13.0%. The remaining seven neoplasms in the combined data comprised myoepithelioma, cystadenoma and sialadenoma papilliferum.

**Conclusions:**

A relatively long duration (2 years) of a submucosal mass in a minor salivary gland-bearing area with or without bone involvement occurring in a child or adolescent should raise the question of a possible salivary gland neoplasm. A pleomorphic adenoma is the most common benign salivary gland neoplasm in the first and second decade of life. Complete surgical excision affords the best chance of preventing recurrence for pleomorphic adenomas. The recurrence rate of pleomorphic adenomas with two or more years follow-up is 13.0%. Other types of minor salivary gland neoplasms are exceedingly rare and therefore data is sparse, precluding any valid conclusions.

## Introduction

Only 3% to5% of all salivary gland neoplasms occur in children and adolescents [1,2]. Two types of neoplasms are found in the salivary glands of pediatric age group patients: neoplasms of epithelial or parenchymal origin and neoplasms of mesenchymal or interstitial origin. The vast majority of the mesenchymal neoplasms occurring in the parotid gland are vasoformative, that is, hemangiomas [1], while the most common types of salivary gland neoplasms of epithelial (parenchymal) origin are pleomorphic adenomas and mucoepidermoid carcinomas.

Most of these epithelial neoplasms are found in the parotid gland; only a limited number of cases occurring in the minor glands of children and adolescents have been well-documented [2]. In fact, a review of the literature revealed only 40 well-documented cases of benign minor salivary gland tumors in this age group [3-28]. Therefore, the purpose of this retrospective analysis was to investigate the clinical features and biologic behavior of a series of benign epithelial minor salivary gland neoplasms occurring in children and adolescents, aged 19 months to 18 years.

## Case presentation

### Prevalence

A total of nine benign minor salivary gland neoplasms were found in patients aged below 19 years out of a total of 396 minor salivary gland neoplasms accessioned over a 35-year period. This is a prevalence of 2.3% in the Louisiana State University School of Dentistry (LSUSD) material. The neoplasms consisted of eight pleomorphic adenomas (PA) and one cystadenoma.

### Summary of findings

A search of the English-language literature revealed 34 PAs [3-23], four myoepitheliomas [25-28], one cystadenoma [7], and one sialadenoma papilliferum [24]. PAs were found to be by far the most commonly occurring benign lesions of minor salivary glands in this age group. A summary of the demographics, clinical findings, treatment and follow-up of the well-documented benign minor salivary gland neoplasms in the literature, in addition to the nine new cases from LSUSD, is shown in Additional file 1: Tables S1 and S2, for a total of 49 cases.

### Pleomorphic adenomas

**Figure 1:**
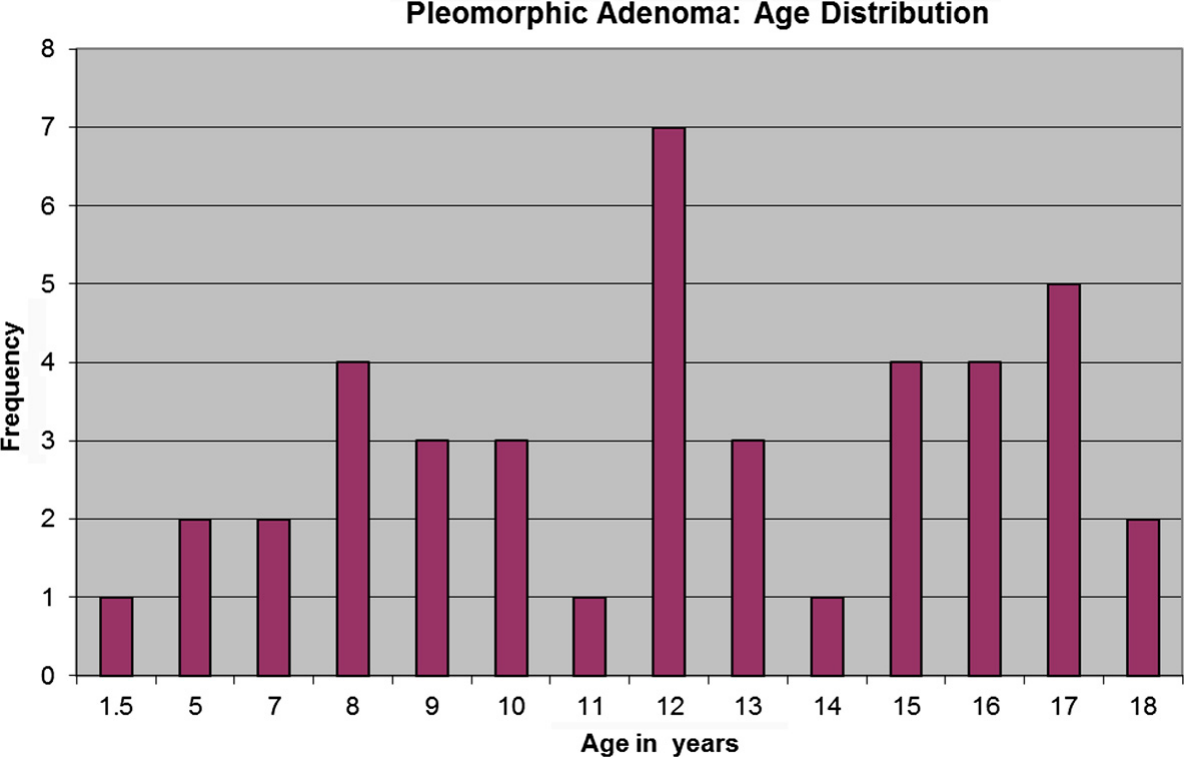
Age distribution of patients with pleomorphic adenoma arising from minor salivary glands

Data for the combined 42 PAs are in Additional file 1: Table S1. The age range was 19 months to 18 years, with a mean of 12 years; 35.7% of the tumors occurred in patients 10 years of age or younger. There was a marked peak in incidence at age 12 years and then again in the later teenage years (Figure 1). The female-to-male ratio was calculated to be 2.8:1. Race was not stated for 21 patients (50%); for the cases reporting race there was essentially an equal distribution between black and white patients. Of the 42 neoplasms, 29 (69.1%) occurred in the minor salivary glands of the hard and/or soft palate, six (14.3%) in the upper lip, four (9.5%) in the buccal mucosa, and three (7.1%) in the tongue. Seven of the palatal neoplasms caused pressure erosion, a smooth depression, or perforation of the under lying bone. One of these palatal neoplasms perforated the palatal bone and extended into the nasal cavity. The majority of PAs were painless, firm submucosal masses or nodules ranging in size from 0.5cm to 5.0cm with a mean of 2.4cm. Sixteen of the 42 cases had reliable patient history reporting a 2.1 year duration of the tumor before definitive diagnosis.

Twenty-four cases with follow-up information of at least 2 years were treated by local surgical excision. Follow-up ranged from 2 to 39 years (mean 9.2 years). Recurrences were reported in three cases for a recurrence rate of 13.0%. The three recurrent cases were all on the hard palate and recurred 2 years, 3 years, and 5 years after the initial surgery.

### Other lesions

Data for the two cystadenomas, one sialadenoma papilliferum and the four myoepitheliomas are presented in Additional file 1: Table S2.

## Discussion

We attempted to outline the clinical features and biologic behavior of benign neoplasms arising from minor salivary glands in children and adolescents. It is acknowledged that there are limitations to the interpretation of data from a retrospective study such as this. For example, the clinical findings and other pertinent information recorded on submitted pathology request forms are unfortunately highly variable and often lack complete information and the same is true for reported cases.

While more pediatric cases have been reported cumulatively in other published salivary gland series, any correlation between patient-specific details and the clinical lesion cannot be made. The approach of our study limits the number of accepted cases, but yields more clinically relevant information. The patients in the LSUSD series were only from the state of Louisiana, thus representing patients from a single geographic area and a single institution experience. The cases accepted from the literature review represent a more global pediatric population, including Hispanic and Asian children, who were not represented in our LSUSD series. Since the socioeconomic status of the children was not known in our study, the association of socioeconomic status to the incidence as well as prognosis of the lesions could not be explored.

Epithelial neoplasms originating in the minor salivary glands account for approximately 15% of all salivary gland neoplasms [29,30]. In the current LSUSD series, 2.3% of the benign epithelial minor salivary gland neoplasms occurred in patients below 19 years of age, which is in close agreement with the series of Waldron *et al*. and Kusama *et al*. who found an incidence of 3.1% and 4.6%, respectively [30,31].

A PA is composed of a wide spectrum of epithelial and mesenchymal tissue derived from cells with ductal and myoepithelial features and it is by far the most common salivary gland neoplasm in children [1,32]. Although it is commonly stated that neoplasms of minor salivary glands rarely occur prior to the second decade [33], we found that, when combining the well-documented literature cases with our LSUSD cases, 28.6% of PAs arising from minor salivary glands in children occurred during the first decade of life. Cumulative data assessment revealed a female gender predilection of 2.8:1 and a predilection for the hard and/or soft palate (69.1%).

Information regarding the biologic behavior of minor salivary gland PAs in children and adolescents is, in general, underprovided. The risk of recurrence of PAs has been speculated to be lower if arising from the minor salivary glands rather than the major glands [34]. In fact, Chau and Radden reported follow-up on 27 intra-oral PAs in patients over a wide age range (second to eighth decade) treated by excisional biopsy and found no recurrences (mean follow-up period 5.3 years), including in two neoplasms which were incompletely excised and two where the tumor extended to the surgical margins [35]. Additionally, Budnick reported a PA in the upper lip of a 17-year-old black woman which was incompletely excised and had no evidence of recurrence on 3 years of follow-up [7]. The recurrence rate of PAs in minor salivary glands of children in our study was 13.0%, which is of clinical significance. This recurrence rate was recorded from 24 well-documented pediatric and adolescent PAs with an average follow-up duration of 9.2 years. The three recurrent PAs involved the hard palate, with one causing bone resorption, one with no bone involvement [19] and one case did not state if there was bone involvement [3]. The assessment for possible causes of recurrence in the three cases varied. The size of the lesion was recorded in two of the three recurrent cases; both were 2.0cm or less in diameter. With regards the surgical margins of these recurrent cases, Byars *et al*. [3] did not comment on the state of the surgical margins, Shaaban *et al*. [19] reported the surgical margins in the definitive excision were free of tumor and, in the LSUSD case, the margins could not be definitively determined as treatment consisted of tumor excision with curettage of the involved palatal bone, making assessment difficult to evaluate with certainty.

In the cases with no recurrence, the surgical margins were not described with any degree of certainty, making it impossible to adequately explore margin involvement as an absolute predictor of recurrence. However, in four of the newly reported LSUSD cases with follow-up of 8 months, 5 years, 8 years and 24 years, the surgical margins were free of tumor. Of those, the case that was followed for 5 years had a focus of tumor in the capsule, but did not recur.

A myoepithelioma is a benign tumor composed of various proportions of plasmacytoid, spindle, epithelioid, and clear cells that exhibit myoepithelial differentiation and generally lack ductal differentiation [36]. While some investigators consider it to be a distinct entity, others believe that it represents one end of the spectrum of the PA and is therefore a variant of PA [27,36]. Regardless of its specific classification, it possesses a similar if not identical biologic behavior to a PA [36]. There are too few cases reported to define its characteristics in the first and second decade of life. However, based on four cases, it does appear to possess similar clinical characteristics and biologic behavior to a PA ( Additional file 1: Table S2).

Minor salivary gland cystadenomas are rare benign well-circumscribed tumors composed of cystic and papillary structures lined with cuboidal or columnar ductal epithelium that appears benign on histology [30]. The average age of incidence is 57 years; they are exceedingly rare below the age of 20 years. The youngest patient reported to have a cystadenoma was 12 years old [7]. Lastly, a sialadenoma papilliferum is a benign exophytic papillary tumor arising from the salivary gland duct. It too is rare below the age of 20 years with only one case reported, in the upper lip of an 18-year-old man [24].

Although minor salivary gland tumors are decidedly uncommon in the first two decades of life, they should be considered in the clinical differential diagnosis for any submucosal nodule or mass in a salivary gland-bearing area. In this study, the average duration of 16 PAs with reliable historical information was 2.1 years before definitive diagnosis. As with any benign or malignant tumor, early definitive diagnosis and appropriate treatment afford a better chance for a cure. This is especially important for minor salivary gland tumors because a high percentage are malignant [1,2].

## Conclusions

The following may be concluded from the results of this study regarding benign epithelial (parenchymal) minor salivary gland tumors in children and adolescents (ages 19 months to 18 years):

1. The prevalence of benign epithelial minor salivary gland tumors in the LSUSD series was 2.3%.

2. Minor salivary gland tumors are more common in female patients.

3. Minor salivary gland tumors have a high predilection for the hard and/or soft palate and have been well-documented in lesser numbers in the upper lip, buccal mucosa, and tongue.

4. Minor salivary gland tumors involving the hard palate may resorb underlying bone.

5. The long duration of a painless submucosal mass in a salivary gland-bearing area does not preclude a minor salivary gland neoplasm.

6. The recurrence rate of minor salivary gland PAs in the first two decades of life is low (13.0%) based on this review of 24 well-documented cases with two or more years follow-up.

7. Complete surgical excision is the most important factor in preventing recurrence of a benign minor salivary gland neoplasm.

8. Long-term clinical follow-up of at least 5 years and the possibility of recurrence should be discussed with the patient and parents when benign minor salivary gland tumors are diagnosed in pediatric and adolescent patients.

## Methods

The archived oral pathology cases from the LSUSD Division of Oral and Maxillofacial Pathology diagnosed as benign salivary gland tumors from January 1 1969 to December 31 2004 were reviewed and patients aged below 19 years with any type of intra-oral benign minor salivary gland neoplasm were selected. The search focused on all types of benign minor salivary gland neoplasms in this age group. All cases consisted of hematoxylin and eosin-stained microscopic slides and the accompanying pathology request form. No cases were rejected for lack of detailed clinical or treatment information. There were no missing specimens or reports in the LSUSD archives. All of the cases in this study were submitted by oral and maxillofacial surgeons in private practice in the state of Louisiana with the exception of one case, which was from the LSUSD Department of Oral and Maxillofacial Surgery. The Louisiana State University Health Sciences Center Institutional Review Board (LSUHSC IRB) approved the research protocol (IRB #6450). Demographic, clinical, and treatment information was recorded for each patient. All of the microscopic diagnoses were made by American Board of Oral and Maxillofacial Pathology-certified pathologists. Additionally, the microscopic diagnosis of each case was reviewed and verified by one of the authors (RBB), an American Board of Oral and Maxillofacial Pathology-certified pathologist, using the diagnostic criteria for PAs and cystadenomas set forth by Ellis and Auclair [36]. A search was performed of the English-language medical and dental literature for well-documented minor salivary gland neoplasms in the pediatric and adolescent age group. It should be noted that, although numerous investigators have published series of salivary gland neoplasms, they provide an age range only and do not correlate the age of the patient to the location of the lesion or offer other demographic and clinical information. Some of these series did indicate that at least one patient was in the first or second decade of life; however, we did not include them in this study because they lacked the adequate detail we required.

## Consent

This study was approved by the LSUHSC IRB (#6450), which oversees and approves all research projects conducted on humans, and also ensures that all research conducted meets ethical standards. The IRB approval obtained for this research project exempted obtaining individual consents from each of the patients on the grounds that it would be virtually impossible to get meaningful data in a retrospective study such as this if authorization is required; some of the pathology reports are up to 20 years old, dentists have lost contact with patients, and dentists periodically purge records, destroying all contact data. The research could not be practically conducted if a consent from each individual patient was required.

## Competing interests

The authors declare that they have no competing interests.

## Authors’ contributions

PR and RBB reviewed the clinical and histopatholgic data from the selected cases and the literature, and analyzed the data. RBB reviewed histopathologic microslides and confirmed the diagnosis for the cases from the LSUSD series. Both authors read and approved the final manuscript.

## Supplementary Material

Additional file 1 **Table S1.** Pleomorphic adenoma. Tabulation of patient demographics, lesion site, duration, bone involvement, treatment and follow-up information for pleomorphic adenoma. **Table S2.** Benign salivary gland tumors (non-PA type). Tabulation of patient demographics, lesion site, duration, bone involvement, treatment and follow-up information for cystadenoma, sialadenoma papilliferum, myoepithelioma, myoepithelioma plasmacytoid variant. Click here for file

## References

[B1] Luna MA Batsakis JG el-Naggar AK Salivary gland tumors in children Ann Otol Rhinol Laryngol 1991 100 869 871 1659302 10.1177/000348949110001016

[B2] Flaitz CM Mucoepidermoid carcinoma of the palate in a child Pediatr Dent 2000 22 292 293 10969433

[B3] Byars LT Ackerman LV Peacock E Tumors of salivary gland origin in children: a clinical pathologic appraisal of 24 cases Ann Surg 1957 146 40 51 10.1097/00000658-195707000-00005 13435700 PMC1451105

[B4] Crawford WH Jr Guernsey LH Pleomorphic adenoma of the palate: report of a case Oral Surg Oral Med Oral Pathol 1967 23 116 126 10.1016/0030-4220(67)90494-X 4288956

[B5] Galich R Salivary gland neoplasms in childhood Arch Otolaryngol 1969 89 878 882 10.1001/archotol.1969.00770020880015 4306739

[B6] Buehrle R Friedberg J Mixed salivary gland tumor of the palate in a child Arch Otolaryngol 1972 96 163 164 10.1001/archotol.1972.00770090237015 4343138

[B7] Budnick SD Minor-salivary-gland tumors in children ASDC J Dent Child 1982 49 44 47 6948837

[B8] Yamamoto H Fukumoto M Yamaguchi F Sakata K Oikawa T Pleomorphic adenoma of the buccal gland in a child Int J Oral Maxillofac Surg 1986 15 474 477 10.1016/S0300-9785(86)80041-2 3018105

[B9] McIlveen LP Sharp HK Schuman NJ Pleomorphic adenoma of a minor salivary gland: report of a case Quintessence Int 1987 18 211 213 3033730

[B10] Lack EE Upton MP Histopathologic review of salivary gland tumors in childhood Arch Otolaryngol Head Neck Surg 1988 114 898 906 10.1001/archotol.1988.01860200082024 2839210

[B11] Rogers TR Johnson JV Newland JR Pleomorphic adenoma of the anterior tongue in a 12-year-old girl J Oral Maxillofac Surg 1989 47 89 90 2536086 10.1016/0278-2391(89)90134-1

[B12] Fonseca I Martins AG Soares J Epithelial salivary gland tumors of children and adolescents in southern Portugal: a clinicopathologic study of twenty-four cases Oral Surg Oral Med Oral Pathol 1991 72 696 701 10.1016/0030-4220(91)90014-4 1667430

[B13] Austin JR Crockett DM Pleomorphic adenoma of the palate in a child Head Neck 1992 14 58 61 10.1002/hed.2880140113 1320597

[B14] Noghreyan A Gatot A Maor E Fliss DM Palatal pleomorphic adenoma in a child J Laryngol Otol 1995 109 343 345 7782697 10.1017/s0022215100130105

[B15] López-Cedrún JL Gonzalez-Landa G Birichinaga B Pleomorphic adenoma of the palate in children: report of a case Int J Oral Maxillofac Surg 1996 25 206 207 10.1016/S0901-5027(96)80031-2 8872225

[B16] de Courten A Lombardi T Samson J Pleomorphic adenoma of the palate in a child: 9-year follow-up Int J Oral Maxillofac Surg 1996 25 293 295 10.1016/S0901-5027(06)80060-3 8910116

[B17] Chen YK Lin LM Lin CC Yan YH Palatal pleomorphic adenoma in a child with osteoid formation: report of case ASDC J Dent Child 1998 65 209 211 9668952

[B18] Bayles SW Todd NW Muller S Rabkin D Pleomorphic adenoma of the pediatric tongue Otolaryngol Head Neck Surg 1999 120 934 936 10.1016/S0194-5998(99)70341-4 10352454

[B19] Shaaban H Bruce J Davenport PJ Recurrent pleomorphic adenoma of the palate in a child Br J Plast Surg 2001 54 245 247 10.1054/bjps.2000.3536 11254420

[B20] Jorge J Pires FR Alves FA Perez DE Kowalski LP Lopes MA Almeila OP Juvenile intraoral pleomorphic adenoma: report of five cases and review of the literature Int J Oral Maxillofac Surg 2002 31 273 275 10.1054/ijom.2002.0206 12190133

[B21] Daniels JS Ali I Al Bakri IM Sumangala B Pleomorphic adenoma of the palate on children and adolescents: a report of 2 cases and review of the literature J Oral Maxillofac Surg 2007 65 541 549 10.1016/j.joms.2006.08.005 17307605

[B22] Lotufo MA Junior CA Mattos JP Franca CM Pleomorphic adenoma of the upper lip in a child J Oral Sci 2008 50 225 228 10.2334/josnusd.50.225 18587216

[B23] Dhanuthai K Sappayatosok K Kongin K Pleomorphic adenoma of the palate in a child: a case report Med Oral Patol Oral Cir Bucal 2009 14 E73 E75 19179953

[B24] Mahajan D Khurana N Setia N Sialadenoma papilliferum in a young patient: a case report of review of the literature Oral Surg Oral Med Oral Pathol Oral Radiol Endod 2007 103 e51 e54 17321439 10.1016/j.tripleo.2006.01.012

[B25] Kahn LB Schoub L Myoepithelioma of the palate. Histochemical and ultrastructural observations Arch Pathol 1973 95 209 212 4346850

[B26] Nesland JM Olafsson J Sobrinho-Simoes M Plasmacytoid myoepithelioma of the palate. A case report with ultrastructural findings and review of the literature J Oral Pathol 1981 10 14 21 10.1111/j.1600-0714.1981.tb01243.x 6259307

[B27] Lins JE Gnepp DR Myoepithelioma of the palate in a child Int J Pediatr Otorhinolaryngol 1986 11 5 13 10.1016/S0165-5876(86)80022-2 2423468

[B28] Perez DE Lopes MA de Almeida OP Jorge J Kowalski LP Plasmacytoid myoepithelioma of the palate in a child Int J Pediatr Dent 2007 17 223 227 10.1111/j.1365-263X.2006.00785.x 17397468

[B29] Eveson JW Cawson RA Tumours of the minor (oropharyngeal) salivary glands: a demographic study of 336 cases J Oral Pathol 1985 14 500 509 10.1111/j.1600-0714.1985.tb00522.x 2991488

[B30] Waldron CA el-Mofty SK Gnepp DR Tumors of the intraoral minor salivary glands: a demographic and histologic study of 426 cases Oral Surg Oral Med Oral Pathol 1988 66 323 333 10.1016/0030-4220(88)90240-X 2845326

[B31] Kusama K Iwanari S Aisaki K Wada M Ohtani J Itoi K Hanai K Shimizu K Komiyama K Kudo I Moro I Intraoral minor salivary gland tumors: a retrospective study of 129 cases J Nihon Univ Sch Dent 1997 39 128 132 10.2334/josnusd1959.39.128 9354027

[B32] Seifert G Okabe H Caselitz J Epithelial salivary gland tumors in children and adolescents. Analysis of 80 cases (Salivary Gland Register 1965–1984) ORL J Otorhinolaryngol Relat Spec 1986 48 137 149 10.1159/000275859 3714196

[B33] Mehta D Willging JP Pediatric salivary gland lesions Semin Pediatr Surg 2006 15 76 84 10.1053/j.sempedsurg.2006.02.004 16616310

[B34] Neville BW Damm DD Allen CM Bouquot JE Oral and Maxillofacial Pathology 2009 3 St. Louis: Saunders Elsevier 480

[B35] Chau MNY Radden BG A clinical-pathological study of 53 intra-oral pleomorphic adenomas Int J Oral Maxillofac Surg 1989 18 158 162 10.1016/S0901-5027(89)80116-X 2474620

[B36] Ellis GL Auclair PL Benign epithelial neoplasms Tumors of the salivary glands 1996 Washington, DC: Armed Forces Institute of Pathology 57 68 Rosai J (Series Editor) Atlas of tumor pathology: series 3, fascicle 17

