# Supplementary material for: A clinical analysis of nine new pediatric and adolescent cases of benign minor salivary gland neoplasms and a review of the literature
Source: J Med Case Rep. 2012 Sep 11;6:287. doi: 10.1186/1752-1947-6-287 (PMC3485090; doi:10.1186/1752-1947-6-287)
Supplement: Additional file 1 — Table S1. Pleomorphic adenoma. Tabulation of patient demographics, lesion site, duration, bone involvement, treatment and follow-up information for pleomorphic adenoma. Table S2. Benign salivary gland tumors (non-PA type). Tabulation of patient demographics, lesion site, duration, bone involvement, treatment and follow-up information for cystadenoma, sialadenoma papilliferum, myoepithelioma, myoepithelioma plasmacytoid variant. [file 1752-1947-6-287-S1.doc]

**Table S1. Pleomorphic Adenomas**

| **Authors** | **Age**  **(Years)** | **Race** | **Gender** | **Site** | **Duration** | **Size** | **Bone Involvement** | **Treatment** | **Follow-Up** |
| --- | --- | --- | --- | --- | --- | --- | --- | --- | --- |
| Byars, et al.[3] | 9 | NS** | F^+^ | Hard palate | NS | NS | NS | -Excision  -Wide excision | - Recur 5 y  -NED 28 y |
| Byars, et al.[3] | 7^+^ | NS** | F^+^ | Hard palate | NS | NS | NS | Surgical excison | NED 7 y |
| Crawford and Guernsey[4] | 8 | W | F | L Hard & Soft palate | ~ 3 weeks | 2.5 cm | NO | Wide local excision | 1 yr NED |
| Galich[5] | 12 | NS | F | Hard/Soft palate extending into pterygoid fossa | 1½ y | 4.5 cm | YES | Excision pterygoid fossa with partial resection inferior maxilla & palate | NED 1½ y |
| Buehrle and Friedberg[6] | 13 | NS | F | R Hard & Soft palate | 2 days | 4.0 cm | YES small area of bony erosion | Surgical excision | 4 months NED |
| Budnick[7] | 12 | B | F | Hard/Soft palate | 1 y | 3.0 cm | NS | “shelled out” | NS |
| Budnick[7] | 13 | B | M | Upper lip | NS | 1.0 cm | -- | Total excision with margins | NED 3y |
| Budnick[7] | 8 | B | F | Buccal mucosa | 3-4 months | 0.5 cm | -- | Excision w margins | NED 3 y |
| Budnick[7] | 5 | NS | M | Buccal mucosa | NS | 4.0 cm | -- | Total excision | NS |
| Budnick[7] | 12 | B | F | Hard palate | NS | NS | NS | Excision w margins | NED 2 y |
| Budnick [7] | 17 | B | F | Upper lip | “Since a baby” | 2.0 cm | -- | Incompletely excised | NED 3 y |
| Budnick[7] | 16 | W | F | Hard palate | NS | 2.0 cm | NS | Excised w margins | NED 20 y |
| Yamamoto et al.[8] | 9 | A | F | Buccal mucosa | 1 y | 1.5 cm | -- | Extirpated | NED 1 y |
| McIlveen et al.[9] | 7 | B | F | Hard palate | NS | 2.0 cm | NS | Excisional Bx | NS |
| Lack and Upton[10] | 10^++^ | NS | M | Soft palate | NS | 2.0 cm | NS | Local resection w + margin followed by re-excision | NED 5 y |
| Rogers et al.[11] | 12 | Hispanic | F | Anterior Tongue | 9 months | 2.0 cm | ---- | Surgical excision | 1 month NED |
| Fonseca et al.[12] | 16 | NS | F | Soft palate | NS | NS | NS | NS | NED 1 y |
| Fonseca et al.[12] | 8 | NS | F | Soft palate | NS | NS | NS | NS | NS |
| Austin and Crockett[13] | 10 | NS | F | Hard palate | 3 weeks | 3.0 cm | Bone: No | Surgical excison | NED 1 y |
| Noghreyan et al.[14] | 8 | NS | F | Hard palate | 2 months | 3.0 cm | NO | Excised w 0.5cm margins down to bone leaving periosteum alone | NED 1½ y |
| Lopez-Cedrun et al.[15] | 16 | NS | M | Hard palate | “2 weeks” | 5.0 cm | NO | Enucleation & curettage of underlying bone | NED 3½ y |
| de Courten  et al.[16] | 10 | W | F | Hard palate | 6 weeks | 2.3 cm | Yes – cupping out | Excision of tumor & periosteum & curettage of bone | NED 9 y |
| Chen et al.[17] | 15 | A | F | R Hard Palate | 4 y | 5.0 cm | NO | Surgical removal | 2 yrs NED |
| Bayles et al.[18] | 19 months | NS | F | R Tongue | 6 weeks | 3.0 cm | ---- | Wide local excision | 16 mos NED |
| Shaaban et al.[19] | 9 | NS | M | Hard palate | “4 days” | 2.0 cm | No bone involve in initial or recurrence | - Wide excision  - Wide excision | Recur 2 y  NED 1 y |
| Jorge et al.[20] | 11 | NS | M | Soft palate | 2 y | 3.0 cm | NO | Surg excision | NED 9 y |
| Jorge et al.[20] | 15 | NS | F | Upper lip | 2 months | 1.0 cm | -- | Surg excision | NED 5 y |
| Jorge et al.[20] | 17 | NS | F | Hard palate | 4 y | 3.0 cm | NO | Surg excision | NED 23 y |
| Jorge et al.[20] | 18 | NS | F | Upper lip | 1 y | 1.0 cm | -- | Surg excision | NED 39 y |
| Jorge et al.[20] | 18 | NS | F | Tongue | 8 y | 1.0 cm | -- | Surgical excision | NED 25 y |
| Daniels et al.[21] | 5 | NS | M | L Hard palate | 20 days | 2 cm | Bone resorption | Surg excision | NED 3 y 10 months |
| Daniels et al.[21] | 16 | NS | M | L Hard palate | 5 months | 1.5 cm | Bone erosion | Surg excision | NED 4 y |
| Lotufu et al.[22] | 12 | NS | M | L Upper lip | 1 y | 2.0 cm | No | Surg excision | NED 1y |
| Dhanuthai et al.[23] | 13 | NS | F | L Hard Palate | 3 y | 1.5 cm | No | Surg excision | NED 8y |
| LSUSD | 15 | W | F | R Hard palate | 1 y | 1.8 cm | NS | Excisional Bx | Unknown |
| LSUSD | 14 | W | F | L Upper lip | 1 y | 1.7 cm | -- | Excisional Bx | NED 24 y |
| LSUSD | 17 | B | F | L Hard palate | 3 y | NS | NS | Incisional Bx | Lost to F/U |
| LSUSD | 17 | W | F | L Hard palate | “Unknown” | 1.3 cm | -Yes: bone resorption  -Recur F/U at 16 months x-ray bone WNL | - Excision w bone curettage  -Excision | -Recur 3 y  -NED 16 months |
| LSUSD | 15 | W | F | L Soft palate | NS | 1.5 cm | NS | Excisional Bx | NED  8y 6 months |
| LSUSD | 12 | W | M | Hard palate | 5 months | 2.1 cm | Yes palatal perforation into nose | Surg exc w periph ostectomy & exc nasal mucosa | 3 weeks |
| LSUSD | 12 | B | M | R Soft palate | 3 months | 5.0 cm | No | Soft Palatectomy via a  Weber-Ferguson | NED  8 months |
| LSUSD | 17 | W | F | L Buccal mucosa | 1 month | 1.2 cm | -- | Excisional Bx | NED 5 y |

**One patient in Byars et al.^3^ study was black but they did not say which case.

+Age and gender according to Austin and Crockett’s^13^ article

++patient had surgery @ the age of 3 years & chemotherapy & radiation @ the age of 2 years for Wilms' tumor prior to development of PA

NS – Not stated

NED – No evidence of disease

LSUSD, Louisiana State University School of Dentistry; B, black; W, white

**Table S2. Benign Salivary gland tumors (non-PA type)**

| **Authors** | **Age** | **Race** | **Gender** | **Site** | **Duration** | **Size** | **Bone Involvement** | **Treatment** | **F/U** | **Diagnosis** |
| --- | --- | --- | --- | --- | --- | --- | --- | --- | --- | --- |
| Budnick[7] | 12 | B | F | Hard Palate | 3-4 months | 1.0 cm | NS | NS | NS | Cystadenoma |
| LSUSD | 17 | W | F | Floor of Mouth | NS | 0.6 cm | ___ | Incisional Biopsy | Unknown | Cystadenoma |
| Mahajan et al.[24] | 18 | NS | M | R Upper lip buccal sulcus | 12 y | 0.8 cm | ___ | Surgical Excision | Did not recur, but no timeline given | Sialadenoma papilleferum |
| Kahn and Schoub[25] | 17 | “Mullato” | F | L Hard palate | 2 months | 3.0 cm | NS | Surgical Excision | NS | Myoepithelioma |
| Nesland et al.[26] | 18 | B | F | Soft palate | NS | 1.5 cm | ____ | Surgical Removal | 1 month NED | Myoepithelioma |
| Lins and Gnepp[27] | 8 | W | F | Soft palate | NS | 1.0 cm | ___ | Surgical  Removal | 1 y NED | Myoepithelioma |
| Da Cruz Perez et al.[28] | 13 | B | M | Junction of hard and soft palate | 2 y | 4 cm | No | Surgical excision | 6 y NED | Myoepithelioma plasmacytoid variant |

NS – Not stated

NED – No evidence of disease

B, black; W, white; LSUSD, Louisiana State University School of Dentistry
